# Pregnancy Following Ventrofixation with Improvements in Technique*Author’s abstract of paper read before American Gynecological Society at Boston 24th May.

**Published:** 1898-06

**Authors:** A. Laphorn Smith

**Affiliations:** M.D., M. R. C. S., England; Fellow of the American Gynecological Society; Professor of Clinical Gynecology, Bishop’s University, Montreal; Gynecologist to the Montreal Dispensary; Surgeon-in-Chief of the Samaritan Hospital for Women; Surgeon to the Western General Hospital


### PREGNANCY FOLLOWING VENTROFIXATION WITH IMPROVEMENTS IN TECHNIQUE.*

By A. LAPHORN SMITH, M.D., M. R. C. S., England.

Fellow of the American Gynecological Society; Professor of Clinical Gynecology, Bishop's University, Montreal; Gynecologist to the Montreal Dispensary; Surgeon-in-Chief of the Samaritan Hospital for Women; Surgeon to the Western General Hospital.

His conclusions were based upon about 2,500 cases by 41 operators, including 111 cases of his own, reported in reply to a circular letter of inquiry.

1. That as far as curing retrodisplacement is concerned, whether retroflexion, retroversion, anteflexion with retroversion, also prolapse of the uterus, ventrofixation with two buried silk stitches passing through peritoneum and fascia gives the most reliable results. Failures are unknown when the operation is performed in this way.

2. Ventrofixation should be reserved for cases in which abdominal section is necessary for other reasons, such as detaching of adhesions and the removal of the diseased tubes which caused the adhesions. When it is expected that pregnancy may follow, some other operation should be chosen, because

3. Although pregnancy only followed in 148 cases out of 2,500, still in 30 per cent. of these, or 36, there was pain, miscarriage or difficult labor requiring obstetrical operations.

4. When suspensio-uteri was performed, that is, the uterus attached to the peritoneum, only a few relapses occurred; but on the other hand, the patients were free from pain during pregnancy and the labors were less tedious; neither did they require resort to serious obstetrical operations. The uterus therefore should be suspended rather than fixed to the abdominal wall in all cases in which any part of the ovary is allowed to remain.

5. A third method, it is claimed by some, namely, the intra-abdominal shortening of the round ligaments, is preferable to either ventrofixation or suspensio-uteri. This may be done either by drawing a loop of the round ligament into the loop which ties off the ovary and tube, or in cases in which the latter are removed, simply to detach them from adhesions and shorten the round ligament by drawing up a loop of it and stitching it to itself for a space of about two inches. By this means the round ligament develops as pregnancy advances, and the dragging and pain and other more serious accidents which are present in 30 per cent. of the cases of ventrofixation are certainly avoided.

6. If the uterus is attached to the abdominal wall, the stitches should be kept on the anterior surface but near the top of the fundus; the complications were more frequent when there was too much adversion than was the case when

*Author's abstract of paper read before American Gynecological Society at Boston 24th May.

the anterior surface of the fundus was attached to the abdominal wall.

7. As large surface as possible should be made to adhere, by scarifying both the anterior surface of the fundus and the corresponding surface of the abdominal peritoneum, in which case one buried silk suture will be sufficient to keep the uterus in good position.

8. Several of my correspondents mentioned incidentally that they knew of many cases of pregnancy after Alexander's operation, and that in no case was the pregnancy or labor unfavorably influenced by it. Alexander's operation should therefore be preferred whenever the uterus and appendages are free from adhesions.

9. The results of Alexander's operation are so good that even when there are adhesions it might be well to adopt the procedure of freeing the adhesions by a very small median incision and then shortening the round ligaments by Alexander's method; after which the abdomen should be closed. This could be done without adding more than ½ of 1 per cent. to the mortality, which in Alexander's operation is nil.

